# Standardization of the ethanolic extract of *Crinum latifolium* leaves by two bioactive markers with antiproliferative activity against TGF-β-promoted prostate stromal cells (WPMY-1)

**DOI:** 10.1186/s12906-022-03617-x

**Published:** 2022-05-18

**Authors:** Wisuwat Thongphichai, Tamonwan Uttarawichien, Pithi Chanvorachote, Supaporn Pitiporn, Todsaphol Charoen-ame, Pakakrong Kwankhao, Pasarapa Towiwat, Suchada Sukrong

**Affiliations:** 1 Center of Excellence in DNA Barcoding of Thai Medicinal Plants, Department of Pharmacognosy and Pharmaceutical Botany, Faculty of Pharmaceutical Sciences, Chulalongkorn University, Bangkok, 10330 Thailand; 2 Department of Pharmacology and Physiology, Faculty of Pharmaceutical Sciences, Chulalongkorn University, Bangkok, 10330 Thailand; 3 Center of Excellence in Cancer Cell and Molecular Biology, Faculty of Pharmaceutical Sciences, Chulalongkorn University, Bangkok, 10330 Thailand; 4 Chao Phya Abhaibhubejhr Hospital Foundation, Prachinburi, 25000 Thailand; 5 Innovation and Product Development Center, SCG Packaging Company Ltd, Ratchaburi, 70110 Thailand

**Keywords:** *Crinum latifolium*, Amaryllidaceae, Benign prostatic hyperplasia (BPH), Alkaloids, Standardization, Bioactive markers

## Abstract

**Background:**

*Crinum latifolium* L. (Amaryllidaceae) has been used in Southeast Asian traditional medicine to alleviate the symptoms of benign prostatic hyperplasia (BPH). The pathological mechanism of BPH is associated with the induction of prostate stromal cell proliferation through transforming growth factor-beta (TGF-β). Standardization as well as investigation of the potential anti-BPH activity of *C. latifolium* extract could benefit the further development of BPH-related analyses and provide evidence to support the application of this extract for BPH treatment. This study aimed to standardize and investigate the antiproliferative activity of the ethanolic extract of *C. latifolium* leaves. The major alkaloids isolated from *C. latifolium* were also explored for their potential use as bioactive markers.

**Methods:**

Two major alkaloids were isolated from the ethanolic extract of *C. latifolium* leaves by chromatographic techniques, identified by NMR and MS, and quantified by a validated UHPLC method. Their antiproliferative activity was studied in human prostate stromal cells (WPMY-1) induced by TGF-β. The synergistic effect of combining the two major isolated alkaloids was analyzed by the zero interaction potency (ZIP) model.

**Results:**

Two alkaloids, lycorine (1) and 6α-hydroxybuphanidrine (2), were isolated from the ethanolic leaf extract of *C. latifolium*. A UHPLC method for the quantification of (1) and (2) was developed and validated in terms of linearity, precision, and accuracy. The *C. latifolium* leaf extract contained 0.279 ± 0.003% (1) and 0.232 ± 0.004% (2). The crude extract was more potent than either (1) and (2) alone against TGF-β-treated WPMY-1 cell proliferation. The drug combination study revealed that the greatest synergistic effect of (1) and (2) was achieved at a 1:1 ratio.

**Conclusions:**

The results of this study support the anti-BPH activity of *C. latifolium* in traditional medicine and suggest that these the two isolated alkaloids may promote the efficacy of the *C. latifolium* extract. Additionally, major alkaloids (1) and (2) can be used as bioactive markers for the standardization of *C. latifolium* extracts.

**Supplementary Information:**

The online version contains supplementary material available at 10.1186/s12906-022-03617-x.

## Background

Benign prostatic hyperplasia (BPH) is the most common urinary tract disease observed in elderly men. Approximately half of all men between ages 51 and 60 have BPH, and 90% of men aged 80 and beyond suffer from this condition [1]. BPH is characterized by the overproliferation of both the stromal and epithelial cells surrounding the transitional zone of the prostate gland, which leads to compression and obstruction of the urethra [2]. Subsequently, BPH patients usually have symptoms that could potentially affect their quality of life. If left untreated, the prostate will grow larger and partially or completely block the urethra, which leads to urinary tract infections [3, 4]. The risk factors for BPH are hormonal alterations, inflammation, oxidative stress, aging and metabolic syndrome [5, 6]. These factors elevate the level of multipotent transforming growth factor-beta (TGF-β), which stimulates the overgrowth of prostate stromal cells [7, 8]. Interestingly, it was shown that stromal cells play a crucial role in BPH development [9]. Lifestyle changes, medication, and surgery are optional treatment options for BPH depending on the age, symptoms, and prostate size of the patient. Medicinal therapies, including alfuzosin, finasteride, and tadalafil, are the most common modality to treat BPH [10]. However, drug treatment has various complications, e.g., urinary tract infections, retrograde ejaculation, bleeding, and erectile dysfunction [11, 12]. Thus, the use of an herbal medicine with few or no side effects has gained attention as an alternative method to treat BPH.

**Fig. 1:**
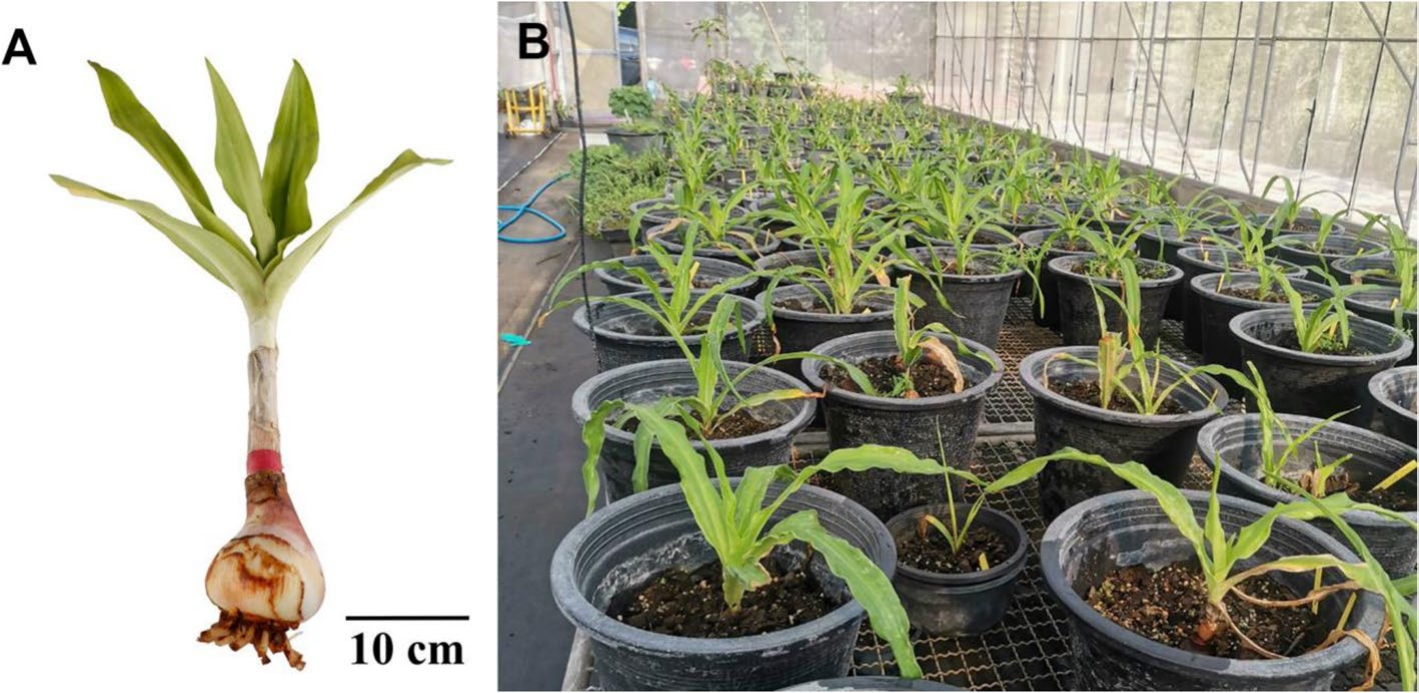
*Crinum latifolium* L. **A** whole plant and **B** plants cultivated at the plant nursery

*Crinum latifolium* L. (Fig. 1), a plant in the Amaryllidaceae family, is naturally distributed throughout Sri Lanka, India, China, Vietnam, Laos, Myanmar, and Thailand [13]. *C. latifolium* has been used in many countries as folk medicine. In Chinese and Vietnamese traditional medicine, *C. latifolium* extract has been used for its antitumor effects [14]. In Thai traditional medicine, *C. latifolium* extract has been used to relieve symptoms related to BPH, including urinary retention [15]. The plant also has antioxidant, antitumor [16], and anti-inflammatory effects [17]. Similar to other plants in the genus *Crinum*, *C. latifolium* is a rich source of alkaloids, including lycorine, which was first isolated as narcissia from *Narcissus pseudonarcissus* L in 1877 [18], and 6α-hydroxybuphanidrine, which was first isolated from *Nerine bowdenii* W [19]. Lycorine is also abundant in many plants in the Amaryllidaceae family and possesses many biological activities, such as antiviral, antitumor, and anti-inflammatory properties [20]. Recent studies have revealed the anti-BPH activity of neferine, an alkaloid obtained from *Nelumbo nucifera* Gaertn, which regulates oxidative stress and apoptosis in BPH [21]. In addition, alkaloid-rich extracts of *Cortex Phellodendri* (dried bark of *Phellodendron amurense* Rupr. or *Phellodendron chinense* C.K.Schneid.) [22] and *Geissospermum vellosii* Allem [23] were suggested to suppress BPH. Hence, *C. latifolium* extract and its alkaloid components may have the potential to possess antiproliferative effects against BPH.

According to traditional medicine, plant extracts are used because they are easily accessible and show greater safety and efficacy than single-compound drugs [24, 25]. However, the main disadvantage of using plant extracts is their uncertain quality due to their chemical complexity. Thus, standardization is essential to guarantee the quality and efficacy of plant extracts. Standardization of plant extracts should ideally rely on certain main components that are easy to analyze, with their quantity representing the efficacy of the extract [26]. In real-world situations, numerous major components have failed to reflect the efficacy of plant extracts, while many therapeutic markers are hard to detect and quantify due to their low quantities [27]. Thus, choosing appropriate chemical constituents for the standardization of plant extracts is crucial.

Although *C. latifolium* has been used in traditional medicine to relieve BPH symptoms, the effects of *C. latifolium* leaf extract on BPH proliferation as well as the method to standardize the extract have not yet been investigated. Thus, this study aimed to standardize the *C. latifolium* leaf extract according to the major alkaloids found in this plant. Additionally, we evaluated the antiproliferative effects of a *C. latifolium* leaf extract and the major isolated alkaloids on specific cell lines related to BPH. Moreover, a drug combination experiment was performed to evaluate the synergistic, additive or antagonistic effects of the combination of the major isolated alkaloids*.*

## Methods

### Chemicals and plant materials

Silica gel of 230–400 and 70–230 mesh size (cat. no. 1.09385 and 1.07734), thin-layer chromatography (TLC) silica gel 60 F_254_ plate (cat. no. 1.05554), high performance thin-layer chromatography (HPTLC) silica gel 60 F_254_ plate (cat. no. 1.05548), chloroform-d1 NMR solvent (cat. no. 102446), dimethyl sulfoxide (DMSO) (cat. no. 1.02952) and glacial acetic acid (cat. no. 1.00063) were purchased from Merck KGaA (Darmstadt, Germany). ACS-grade ammonium acetate (cat. no. 01080) was obtained from Loba Chemie Pvt. LTD. (Maharashtra, India). DMSO-d6 (cat. no. DLM-10–25) was acquired from Cambridge Isotope Laboratories, Inc. (Massachusetts, USA). Sephadex LH-20 (cat. no. 17–0090-01) was obtained from Pharmacia Biotech AB (Uppsala, Sweden). HPLC-grade acetonitrile (cat. no. LC1005), AR-grade chloroform (cat. no. AR1027E) and 98% sulfuric acid (cat. no. AR1193) were garnered from RCI Labscan (Bangkok, Thailand). A 28% ammonia solution (cat. no. 43–2.5L GL) was obtained from APS Chemicals Ltd. (Pontypridd, UK). Ultrapure water was generated with a Barnstead™ MicroPure™ Water Purification System (Thermo Fisher Scientific, Massachusetts, USA, (cat. no. 50132370). Ethanol was received from T.S. Interlab LP (Bangkok, Thailand). Commercial hexane, dichloromethane, ethyl acetate (EtOAc), acetone, and methanol (MeOH) were purchased from a local chemical company and distilled before use. Dulbecco’s modified Eagle’s medium (DMEM) (cat. no. 2230805), 10% fetal bovine serum (FBS) (cat. no. F0804), 1% GlutaMAX (cat. no. 1IVG7-35,050–061), 1% penicillin–streptomycin (cat. no. 1IVG7-15,140–122), 3-(4,5-dimethylthiazol-2-yl)-2,5-diphenyltetra-sodium bromide (MTT) reagents (cat. no. M6494) and liquid chromatography/mass spectrometry (LC/MS) grade methanol (cat. no. 10402824) were acquired from Thermo Fisher Scientific (Massachusetts, USA). TGF-β (cat. no. GF113) was obtained from Merck KGaA (Darmstadt, Germany).

*C. latifolium* were collected from Ratchaburi Province, Thailand. The samples were identified by Associate Professor Thatree Phadungcharoen, a taxonomist at the Faculty of Pharmaceutical Sciences, Chulalongkorn University. A voucher specimen (SS-PharmCU-11–2021) was deposited at the Museum of Natural Medicines, Chulalongkorn University, Bangkok, Thailand.

### Preparation of the *C. latifolium* extract

The fresh leaves of *C. latifolium* were cleaned, chopped, and dried in a hot oven at 50 °C for 24 h. The dried leaves (120 g) were further ground to powder, placed in a fabric bag and macerated with 70% ethanol (1:40 w/v) for 7 d at room temperature with occasional stirring. The extract was then collected and filtered through a cotton wool plug. The plant leaves were re-macerated until exhausted. All collected filtrates were pooled together and evaporated *in vacuo* to obtain 32.16 g of crude ethanolic extract.

### Isolation and characterization of the major alkaloids from *C. latifolium* extract

The crude extract of *C. latifolium* leaves was subjected to a series of SiO_2_ and Sephadex LH-20 columns. Fractions from each column were collected and combined based on TLC observations. Alkaloid-containing fractions were traced by reaction with Dragendorff’s reagent on TLC plates. Isolated alkaloids were analyzed on a Bruker Ascend 400 NMR spectrometer (Massachusetts, USA) to acquire ^1^H-NMR, ^13^C-NMR, COSY, HSQC, HMBC and NOESY correlation spectra. Each NMR sample was prepared by dissolving 7–10 mg of an isolated alkaloid in 0.5 mL of deuterated solvents followed by transfer to an NMR tube (DWK Life Sciences, Mainz, Germany, cat. no. 231700117). To obtain the molecular weights of the isolated alkaloids, each compound was dissolved in LC/MS grade MeOH to a concentration of 50 ppm, and then 20 μL of the solution was directly injected into a Bruker Daltonics micrOTOF II spectrometer (Massachusetts, USA) to obtain mass spectra.

### Standardization of the *C. latifolium* extract by ultrahigh-performace liquid chromatography (UHPLC)

The ethanolic extract of *C. latifolium* leaves was standardized by using two major isolated alkaloids. Chromatographic analysis was conducted using an Agilent 1290 Infinity II UHPLC system in combination with a Zorbax Eclipse Plus C-18 reversed-phase column (150 mm × 4.6 mm, 5 μm; Agilent, California, USA) and a C-18 guard column. The mobile phases were acetonitrile (A) and 1% ammonium acetate solution with 0.3% acetic acid (B); both solutions were filtered through a 0.2 μm nylon filter (Vertical, Bangkok, Thailand, cat. no. 0235–0101) before use. Each *C. latifolium* sample solution was prepared by dissolving 40 mg of the *C. latifolium* leaf ethanolic extract in 2 mL of 3% H_2_SO_4_ and then washing with diethyl ether 3 times. The solution was further basified with 3 mL of 28% ammonia, followed by extraction with chloroform (3 × 3 mL). The chloroform extracts were combined and evaporated *in vacuo*, and then the residue was redissolved in 20% A/B and successively filtered through a ChromPlus® 0.2 μm PTFE syringe filter (Chemplus, Jiangsu, China, cat. no. CPSFPTFE2522NS-B). Reference standard solutions for generating calibration curves were prepared by dissolving the isolated alkaloids in 1 mL of 20% A/B and then filtering the solutions through a 0.2 μm PTFE syringe filter to yield 1.0 mg/mL stock solutions. Working solutions were obtained by serial dilution of the stock solutions with 20% A/B to concentrations of 1, 4, 7, 10, 40, 70, and 100 μg/mL. All solutions were kept at 5 °C until analysis. The chromatographic gradient elution program with A/B was as follows; 8% for 5.5 min, 8–20% for 0.5 min, 20% for 14 min, 20–80% for 5 min, 80% for 5 min, 80–8% for 5 min and 8% for 5 min. The flow rate was 1.0 mL/min, and the injection volume was 10 μL. The experiments were conducted at room temperature (28 °C) and monitored with a diode array detector at 280 nm. Calibration curves were generated by plotting the concentration of seven reference standards versus the areas under the peaks.

### Method validation

Method validation was performed in accordance with the International Council for Harmonisation (ICH) harmonised tripartite guidelines [Q2(R1)]. The method was validated in terms of linearity, precision, accuracy, limit of detection (LOD), and limit of quantitation (LOQ). Linearity was determined using the correlation coefficient (R^2^) of the calibration curve. Precision was divided into intraday and interday precision. For intraday precision, seven known standard solutions were injected three times within one day. Interday precision was examined by injecting the seven known standard solutions one time per day on three consecutive days. The intra- and interday precision values are represented as the relative standard deviation (%RSD) calculated from the peak areas obtained. Accuracy was determined by using the standard addition method. The samples were spiked with three standard concentrations of 10, 20 and 40 μg/mL. Each concentration was analyzed in triplicate, and the percent recovery was calculated. The LOD and LOQ were evaluated from the standard deviation (SD) and slope (S) of each calibration curve. The LOD and LOQ are expressed as (3 × SD)/S and (10 × SD)/S, respectively.

### Cell culture

WPMY-1 cells were purchased from the American Type Culture Collection (ATCC no. CRL-2854™). The cells were cultured in high-glucose DMEM supplemented with 10% FBS, 1% GlutaMax, and 1% penicillin–streptomycin. The cells were maintained at 37 °C in a humidified atmosphere within an incubator that was supplied with 5% CO_2_.

### Cytotoxicity and antiproliferative tests

To determine the maximum nontoxic dose of the tested compounds, WPMY-1 cell viability was investigated by the MTT method [28]. WPMY-1 cells at a density of 1 × 10^3^ cells/well were seeded in 96-well plates and incubated at 37 °C with 5% CO_2_ for 24 h. Cells were then treated with the test compounds or extract for 72 h. Next, serum-free medium containing MTT solution was added for 4 h of incubation, followed by the addition of DMSO. The results were determined by measuring the absorbance at 570 nm with a microplate reader (CALIOSTAR).

The antiproliferative effect of the maximum nontoxic dose of each tested compound was assessed by the MTT method. TGF-β (5 ng/mL) was used to induce WPMY-1 cell proliferation. WPMY-1 cells (1 × 10^3^ cells) were seeded in 96-well plates and incubated at 37 °C with 5% CO_2_ for 24 h. Then, the medium was replaced with serum media that contained 5 ng/mL TGF-β supplemented with 10 µg/mL *C. latifolium* leaf extract or 5 ng/mL isolated alkaloid. Mitomycin C (5 ng/mL) was used as a positive control. After 72 h, WPMY-1 cell proliferation was analyzed with a microplate reader at 570 nm. The experiments were performed in triplicate.

### Drug combination test

The drug combination test was used to assess the combination effect of the two major isolated alkaloids. The experiment was performed in the same manner as the antiproliferative assay. WPMY-1 cells were treated by adding 5 ng/mL TGF-β and the combination of the two major isolated alkaloids at concentrations ranging from 1 to 5 ng/mL and incubating for 72 h prior to analysis with a microplate reader at 570 nm. The results were calculated by the zero interaction potency (ZIP) reference model using the SynergyFinder 2.0 program [29].

### Statistical analysis

All results are presented as the mean ± SD. Analysis of the quantitative UHPLC results was performed using Microsoft Excel 2019 software. The results from the biological assays were analyzed with GraphPad Prism 9 software using one-way analysis of variance (one-way ANOVA) followed by Tukey’s post-hoc test. A *P* value < 0.05 was considered statistically significant.

## Results

### The ethanolic extract of *C. latifolium* leaves inhibits TGF-β-induced WPMY-1 cell proliferation

**Fig. 2:**
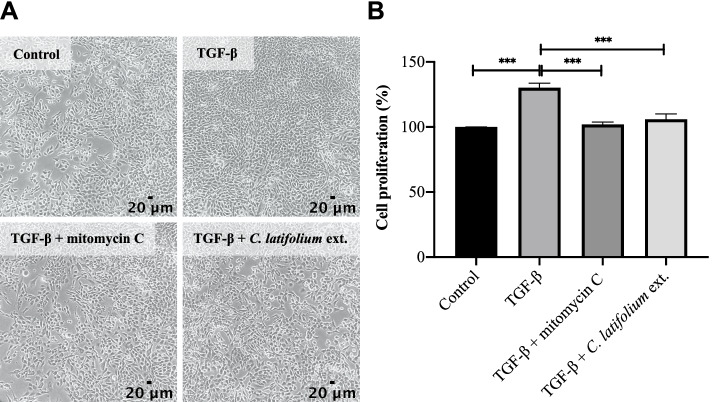
Effects of different treatments on the proliferation of WPMY-1 cells. **A** Images of WPMY-1 cells treated without TGF-β (control), with TGF-β, with TGF-β supplemented with 5 ng/mL mitomycin C, and with TGF-β supplemented with 10 μg/mL *C. latifolium* extract. All images were captured at 100 × magnification; scale bar = 20 µm. **B** Bar graph revealing the percent cell proliferation represented as the means ± SD (***P < 0.001)

The maximum nontoxic concentrations of mitomycin C and *C. latifolium* leaf extract were determined from the cytotoxicity assay to be 5 ng/mL (92.75 ± 3.90% cell viability) and 10 μg/mL (91.55 ± 6.34% cell viability), respectively (Figs. S1 and S2); thus, these concentrations were used on the antiproliferative assay. The optimized TGF-β concentration was 5 ng/mL, which was used to induce WPMY-1 proliferation (Fig. S3). The results showed that treatment with TGF-β alone significantly increased the proliferation of WPMY-1 cells to 130.19 ± 3.44% compared with 100% for untreated cells. However, the number proliferated WPMY-1 cells was dramatically decreased after treatment with *C. latifolium* leaf extract, giving a value similar to that after treatment with the positive control mitomycin C (Fig. 2).

### Lycorine (1) and 6α-hydroxybuphanidrine (2) were isolated from *C. latifolium* extract

**Fig. 3:**
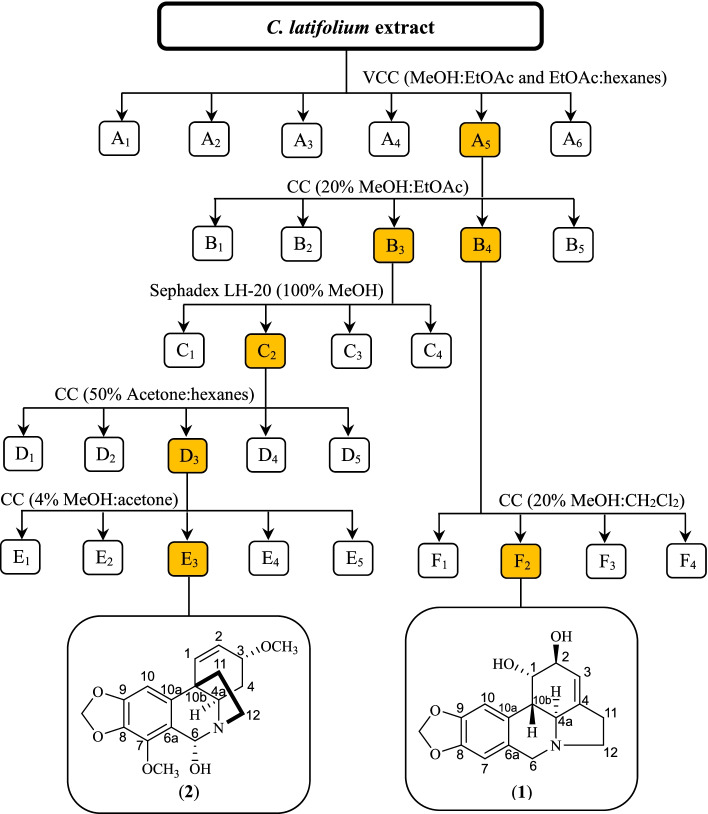
Flowchart for the isolation of the major alkaloids lycorine (**1**) and 6α-hydroxybuphanidrine (**2**) from the *C. latifolium* leaf extract. The alkaloids were identified by TLC sprayed with Dragendorff’s reagent. VCC, vacuum column chromatography; CC, column chromatography

To isolate major alkaloids from *C. latifolium*, the ethanolic extract (32.16 g) was subjected to vacuum column chromatography over SiO_2_ (230–400 mesh) and eluted with gradient systems of hexanes:EtOAc and MeOH:EtOAc. The fractions from the column were collected and combined based on the TLC data to afford fractions A_1_–A_6_ (Fig. 3). Fraction A_5_ (4.62 g), which was proven to contain alkaloids by Dragendorff’s test, was further separated by SiO_2_ (70–230 mesh) column chromatography. The column was eluted with an isocratic 20% MeOH:EtOAc system to obtain subfractions B_1_–B_5_. Subfractions B_3_ and B_4_, which were proven to contain alkaloids, were further separated by SiO_2_ chromatography and Sephadex LH-20 to afford lycorine (1) and 6α-hydroxybuphanidrine (2). The purities of (1) and (2) were proven to be ≥ 95% by using UHPLC analysis.

Lycorine (1): white needles (MeOH); UV (EtOH) λ_max_ (log ε): 235 (3.35) and 292 (3.45) nm (Fig. S4); high-resolution time-of-flight mass spectrometry (HRTOFMS): m/z 288.1231 [M + H]^+^ (calculated for C_16_H_18_NO_4_: 288.1236) (Fig. S5). The ^1^H-NMR spectrum of (1) (Fig. S6) revealed two *para*-aromatic protons (H-1 and H-2) at *δ* 6.80 and 6.68 ppm, respectively. A multiplet at *δ* 5.94–5.95 ppm was ascribed to be a methylenedioxy group. A broad singlet at *δ* 5.37 ppm was identified as olefinic methine proton H-3, while two broad singlets at *δ* 4.27 and 3.97 ppm represented oxygenated methine protons H-1 and H-2, respectively. The ^13^C-NMR spectrum of (1) (Fig. S7) revealed two oxygenated aromatic carbons C-8 and C-9 at *δ* 145.7 and 145.2 ppm, respectively. while the signal at *δ* 100.6 ppm represented a methylenedioxy group. Compound (1) was confirmed to be lycorine by comparison of its ^1^H and ^13^C-NMR spectra with those in a previous report [30] (Table 1).

**Table 1:** ^1^H-NMR (*δ*_H_, *J*) and ^13^C-NMR (*δ*_C_) spectral data obtained in this study for lycorine (**1**) in comparison to those of a previous report (*δ*_H_^a^, *J*^a^, *δ*_C_^a^)

Position	*δ*_H_, mult. (*J*)	*δ*_H_^a^, mult. (*J*^a^)	*δ*_C_	*δ*_C_^a^
1	4.27 brs	4.25 m	70.2	70.3
2	3.97 brs	3.96 m	71.7	71.8
3	5.37 brs	5.35 brs	118.5	118.6
4	-	-	141.7	141.9
4a	2.60 brd (10.3)	2.59 d (10.4)	60.8	60.8
6α	3.32 d (14.2)	3.31 d (14.0)	85.7	85.6
6β	4.01 d (14.2)	4.00 d (14.0)		
6a	-	-	129.8	129.9
7	6.68 s	6.66 s	107.0	107.2
8	-	-	145.2	145.4
9	-	-	145.7	145.8
10	6.80 s	6.79 s	105.1	105.2
10a	-	-	129.6	129.7
10b	2.41 m	2.42 m	40.2	40.2
11	2.45–2.54 m	2.45–2.53 m	28.1	28.2
12α	2.20 q (8.7)	2.19 m	53.3	53.4
12β	3.33 ddd (13.0, 10.1, 4.4)	3.31 ddd (13.0, 10.5, 4.5)		
-OCH_2_O-	5.94–5.95 m	5.90–5.96 m	100.6	100.7
1-OH	4.77 d (4.1)	4.79 m		
2-OH	4.87 d (6.2)	4.91 m		

The chemical shift (*δ*) is represented in ppm

The coupling constant (*J*) is represented in Hz

*δ*_H_^a^, *δ*_C_^a^ and *J*^a^ were obtained from [30]

6α-Hydroxybuphanidrine (2): white solid (MeOH); UV (EtOH) λ_max_ (log ε): 286 (3.05) nm (Fig. S8); HRTOFMS: m/z 332.1495 [M + H]^+^ (calculated for C_18_H_22_NO_5_: 332.1498) (Fig. S9). The ^1^H-NMR spectrum of (2) (Fig. S10) revealed aromatic proton H-10 at *δ* 6.57 ppm, while the signals at *δ* 6.54 and 5.96 ppm represented two *cis*-olefinic protons (H-1 and H-2, respectively, *J*_*cis*_ = 10.0 Hz). A singlet at *δ* 5.24 ppm was determined to be oxygenated benzylic proton H-6β. The methylenedioxy protons appeared as a multiplet at *δ* 5.86–5.89 ppm, while two methoxy groups (7-OCH_3_ and 3-OCH_3_) resonated at *δ* 4.04 and 3.34 ppm, respectively. The ^13^C-NMR spectrum of (2) (Fig. S11) revealed three oxygenated aromatic carbons C-7, C-8 and C-9 at *δ* 142.5, 134.2 and 149.3 ppm, respectively. The signals at *δ* 132.1 and 125.9 ppm were determined to be olefinic methine carbons (C-1 and C-2, respectively). The methylenedioxy moiety appeared at *δ* 100.8 ppm, while two methoxy carbons (7-OCH_3_ and 3-OCH_3_) resonated at *δ* 47.9 and 40.8 ppm, respectively. Compound (2) was identified as 6α-hydroxybuphanidrine by comparison of its ^1^H and ^13^C-NMR spectra with those previously reported in the literature (Table 2) [31].

**Table 2:** ^1^H-NMR (*δ*_H_, *J*) and ^13^C-NMR (*δ*_C_) spectral data obtained in this study for 6α-hydroxybuphanidrine (**2**) in comparison to those of a previous report (*δ*_H_^a^, *J*^a^, *δ*_C_^a^)

Position	*δ*_H_, mult. (*J*)	*δ*_H_^a^, mult. (*J*^a^)	*δ*_C_	*δ*_C_^a^
1	6.54 d (10.0)	6.52 d (10.0)	132.1	131.9
2	5.96 ddd (10.0, 5.1, 1.0)	5.94 ddd (10.0, 5.0, 0.5)	125.9	125.8
3	3.82 *obsc*	3.79 ddd (5.0, 4.0, 1.5)	72.4	72.3
4α	2.10 ddt (13.9, 4.2, 1.5)	2.08 dddd (14.0, 4.0, 1.5, 0.5)	28.0	27.9
4β	1.56 td (13.6, 4.2)	1.54 ddd (14.0, 13.5, 4.0)		
4a	3.84 ddd (13.7, 4.0)	3.84 ddd (13.5, 4.0, 0.5)	56.5	56.4
6α	-	-	85.7	85.6
6β	5.24 s	5.24 s		
6a	-	-	119.3	119.2
7	-	-	142.5	142.5
8	-	-	134.2	134.2
9	-	-	149.3	149.3
10	6.57 s	6.55 s	97.1	97.0
10a	-	-	140.0	139.8
10b	-	-	44.2	44.2
11*endo*	1.91 m	1.90 dddd (12.5, 9.0, 4.5, 0.5)	40.8	40.8
11*exo*	1.88 m	1.84 ddd (12.5, 10.5, 6.0)		
12*endo*	2.81 ddd (13.0, 8.9, 6.1)	2.79 ddd (13.0, 9.0, 6.0)	47.9	47.7
12*exo*	3.33 ddd (13.0, 10.1, 4.4)	3.31 ddd (13.0, 10.5, 4.5)		
3-OCH_3_	3.34 s	3.32 s	56.4	56.3
7-OCH_3_	4.04 s	4.01 s	59.8	59.8
-OCH_2_O-	5.86–5.89 d (1.5)	5.84–5.87 d (1.5)	100.8	100.8

The chemical shift (*δ*) is represented in ppm

The coupling constant (*J*) is represented in Hz

*obsc.* = obscured signal

*δ*_H_^a^, *δ*_C_^a^ and *J*^a^ were obtained from [31]

### Development and validation of a UHPLC method for the standardization of *C. latifolium* extract using lycorine (1) and 6α-hydroxybuphanidrine (2)

**Fig. 4:**
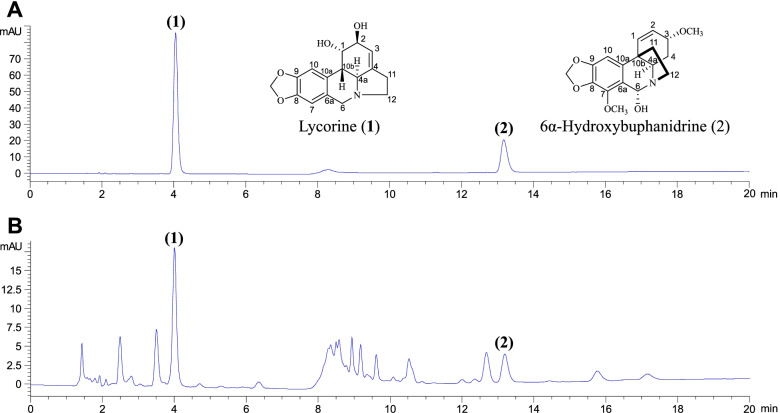
**A** Chromatograms of lycorine (**1**) and 6-hydroxybuphanidrine (**2**) in a standard solution.** B** Chromatogram of the *C. latifolium* leaf extract

A UHPLC method was developed to support simultaneous analysis of (1) and (2). The method was modified from a previous report [32] for optimal separation. After various mobile phase trials, the gradient system of acetonitrile (A) and 1% ammonium acetate with 0.3% acetic acid (B) provided sharp, symmetric peak shapes with a short analysis time and good resolution (Fig. 4). Eight percent A was used from 0–5.5 min to optimize the resolution and retention time of (1), which appeared at 4.0–4.1 min. At 5.5–6.0 min, the polarity was changed from 8–20% A. Twenty percent A was used from 6.0–20.0 min to optimize the separation of (2), which eluted at 13.1–13.2 min. The polarity changes from 20.0–40.0 min were used for column cleaning and equilibration.

**Fig. 5:**
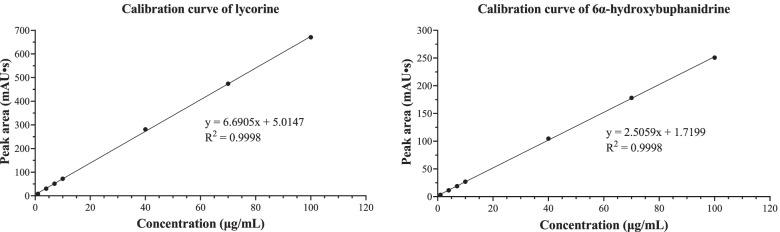
Calibration curves of lycorine and 6α-hydroxybuphanidrine

Method validation was performed to determine reliability. The calibration curves of (1) and (2) showed coefficient of determination (R^2^) values of 0.9998 (Fig. 5). The calculated LOD and LOQ of (1) were 2.713 and 9.042 µg/mL, respectively, while those of (2) were 2.557 and 8.523 µg/mL, respectively (Table 3). The developed method achieved acceptable precision by considering %RSD. The intraday and interday precision %RSD values of (1) were less than 0.552 and 1.977, respectively, whereas these values for (2) were lower than 1.450 and 1.593, respectively. (Tables 4 and 5). Accuracy was established by the standard addition method. The results showed good accuracy, as the percent recoveries were 97.68–105.51% and 93.81–95.99% for (1) and (2), respectively (Table 6).

**Table 3:** Calibration curve, equations and calculated LODs, LOQs, and ranges of quantitation for lycorine and 6α-hydroxybuphanidrine

Parameter	Lycorine	6α-Hydroxybuphanidrine
Regression equation	y = 6.6905x + 5.0147	y = 2.5059x + 1.7199
Coefficient of determination (R^2^)	0.9998	0.9998
Standard deviation of signal (σ)	6.0499	2.1360
Limit of detection (LOD)	2.713 µg/mL	2.557 µg/mL
Limit of quantitation (LOQ)	9.042 µg/mL	8.523 µg/mL
Range of quantitation	9.04–100 µg/mL	8.52–100 µg/mL

**Table 4:** Intraday and interday precision result for lycorine

Exp	Intraday precision			Interday precision		
	Conc. (µg/mL)	Mean ± SD^a^	%RSD^b^	Conc. (µg/mL)	Mean ± SD^a^	%RSD^b^
1	1.218 1.220 1.217	1.218 ± 0.001	0.123	1.256 1.284 1.279	1.273 ± 0.015	1.199
2	4.512 4.528 4.538	4.533 ± 0.018	0.407	4.548 4.559 4.635	4.580 ± 0.047	1.033
3	7.692 7.705 7.632	7.676 ± 0.039	0.508	7.705 7.747 7.954	7.802 ± 0.134	1.713
4	10.90 10.99 10.86	10.89 ± 0.02	0.197	10.89 10.89 11.16	10.98 ± 0.16	1.430
5	42.12 42.09 42.21	42.14 ± 0.06	0.150	42.09 42.50 43.31	42.63 ± 0.62	1.464
6	70.92 71.22 71.65	71.26 ± 0.37	0.515	72.04 73.21 74.92	73.39 ± 1.45	1.977
7	100.21 100.77 101.32	100.77 ± 0.56	0.552	101.32 102.67 104.33	102.77 ± 1.50	1.463

^a^*SD* Standard deviation

^b^*%RSD* Relative standard deviation

**Table 5:** Intraday and interday precision results for 6α-hydroxybuphanidrine

Exp	Intraday precision			Interday precision		
	Conc. (µg/mL)	Mean ± SD^a^	%RSD^b^	Conc. (µg/mL)	Mean ± SD^a^	%RSD^b^
1	1.249 1.273 1.281	1.268 ± 0.017	1.311	1.293 1.261 1.261	0.973 ± 0.006	0.662
2	4.549 4.457 4.521	4.509 ± 0.047	1.043	4.473 4.553 4.525	4.602 ± 0.060	1.593
3	7.339 7.339 7.462	7.380 ± 0.071	0.968	7.602 7.830 7.762	6.608 ± 0.063	0.953
4	10.95 10.65 10.90	10.83 ± 0.16	1.450	10.95 10.91 11.10	10.99 ± 0.10	0.927
5	41.84 41.89 42.29	42.01 ± 0.24	0.582	42.29 43.12 43.55	42.99 ± 0.64	1.491
6	70.63 70.16 71.63	70.81 ± 0.75	1.059	72.03 73.47 73.03	72.84 ± 0.74	1.016
7	99.42 100.21 101.16	100.26 ± 0.87	0.869	101.16 102.58 103.96	102.57 ± 1.40	1.366

^a^*SD* Standard deviation

^b^*%RSD* Relative standard deviation

**Table 6:** Accuracy test results for lycorine and 6α-hydroxybuphanidrine

	Exp	Sample concentration (µg/mL)	Added concentration (µg/mL)	Measured concentration (µg/mL)	Recovery (%) ± SD^a^	%RSD^b^
Lycorine	1	22.32	10.00	32.09	97.68 ± 1.35	1.387
	2	22.32	20.00	42.56	101.21 ± 0.96	0.953
	3	22.32	40.00	64.52	105.51 ± 1.41	1.334
6α-Hydroxy-buphanidrine	1	18.55	10.00	28.15	95.99 ± 1.49	1.556
	2	18.55	20.00	37.18	94.24 ± 1.28	1.358
	3	18.55	40.00	56.07	93.81 ± 0.88	0.811

^a^*SD* Standard deviation

^b^*%RSD* Relative standard deviation

The validated UHPLC method was used to determine the amounts of (1) and (2) in the *C. latifolium* leaf extract; these values were 0.279 ± 0.003% and 0.232 ± 0.004% (w/w), respectively. The quantities of (1) and (2) in the dry leaf sample were calculated to be 0.0748 ± 0.0007% and 0.0621 ± 0.0010% (w/w), respectively.

### Lycorine (1) and 6-hydroxybuphanidrine (2) exhibited antiproliferative effects on TGF-β-treated WPMY-1 cells

**Fig. 6:**
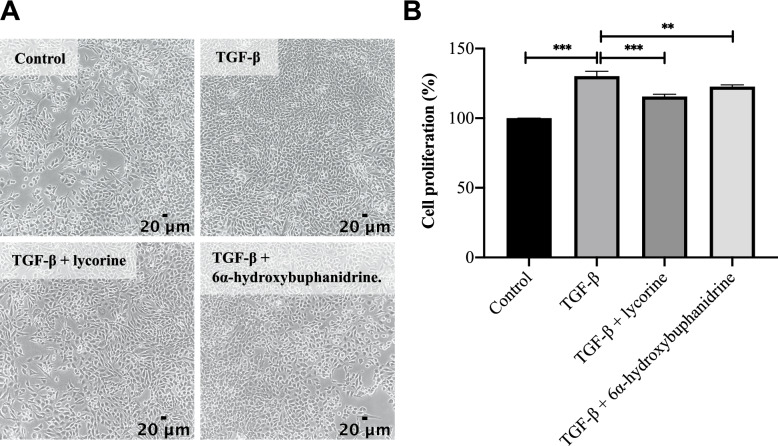
Effects of different treatments on the proliferation of WPMY-1 cells. **A** Images of WPMY-1 cells treated without TGF-β (control), with TGF-β (5 ng/mL), or with TGF-β supplemented with lycorine or 6α-hydroxybuphanidrine (each 5 ng/mL). All images were captured at 100 × magnification; scale bar = 20 µm. **B** Bar graph revealing the percent cell proliferation represented as the means ± SD (**P < 0.01 and ***P < 0.001)

To investigate the potential of (1) and (2) found in the *C. latifolium* leaf extract to act as bioactive compounds, cell viability and antiproliferative assays were conducted with (1) and (2). The maximum nontoxic dose of both (1) and (2) was determined to be 5 ng/mL (89.35 ± 9.22% and 97.26 ± 4.73% cell viability, respectively) (Figs. S12 and S13). The results demonstrated that TGF-β-induced WPMY-1 cell proliferation decreased significantly after treatment with (1) and (2) (Fig. 6). This finding showed that the two major isolated alkaloids, (1) and (2), exhibited antiproliferative activity against TGF-β-treated WPMY-1 cells.

### Lycorine (1) and 6α-hydroxybuphanidrine (2) had additive effects against of TGF-β-treated WPMY-1 cell proliferation

**Fig. 7:**
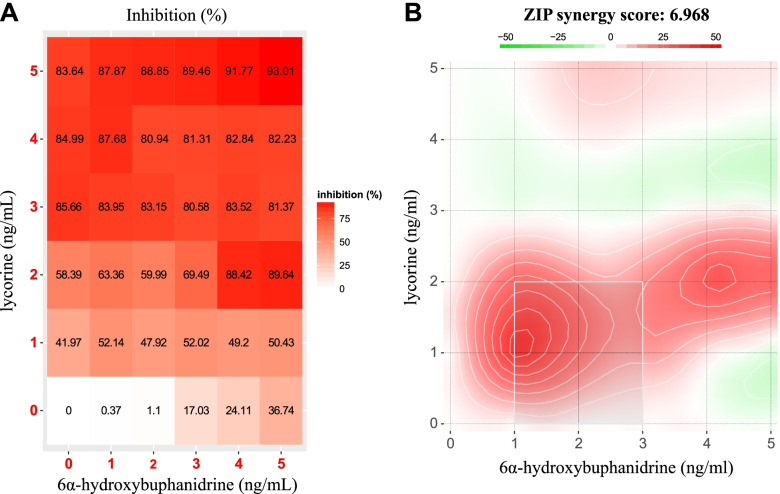
Drug combination study of lycorine (**1**) and 6α-hydroxybuphanidrine (**2**). **A** Dose–response matrix representing the percent inhibition of WPMY-1 cells treated with TGF-β supplemented with (**1**) and (**2**) at concentration ranging from 1–5 ng/mL. **B** Interaction landscape showing the combined effects of (**1**) and (**2**). The red region indicates synergism, the white region shows an additive effect and the green region represents antagonism

The results from the antiproliferative assays with the *C. latifolium* leaf extract and the two major isolated alkaloids revealed that the *C. latifolium* leaf extract had a greater inhibitory effect than each of the individual isolated alkaloids. Thus, a drug combination assay with (1) and (2) was performed to evaluate their potential synergistic effect. The dose–response matrix of (1) and (2) demonstrated that in the absence of (2), the percent inhibition of WPMY-1 cells increased sharply when the concentration of (1) increased from 0–3 ng/mL. However, the percent inhibition nonsignificantly increased when the concentration of (1) was more than 3 ng/mL. Unlike (1), the percent inhibition of WPMY-1 cells significantly increased in a manner that was directly proportional to the concentration of (2) at all tested concentrations (Fig. 7A). An interaction landscape was thus constructed to show the synergistic, additive and antagonistic effects with red, white and green areas, respectively. The results mostly revealed a synergistic effect of the alkaloid combination, and the strongest synergistic effect appeared when the concentrations of (1) and (2) were 1 ng/mL. However, the summary synergy ZIP score was calculated to be 6.968, showing an additive effect of the two compounds (Fig. 7B).

## Discussion

In traditional Thai medicine, *C. latifolium* has been used for the treatment of symptoms related to BPH [15]. In Vietnam, dietary supplement products made from *C. latifolium* were launched and claimed to the relieve symptoms of BPH, but studies on the anti-BPH mechanism of *C. latifolium* are limited. A recent study revealed that in BPH, the proliferation of stromal cells is four times greater than that of epithelial cells [9]. Because the proliferation of stromal cells plays an important role in BPH, prostate stromal cells (WPMY-1 cells), which are a predominant cell type involved in BPH progression, were used in this study. A previous study reported the effects of TGF-β on the proliferation of BPH stromal cells. At low doses, TGF-β promotes prostate stromal cell proliferation, while high doses of TGF-β potently induce cell growth arrest [7]. Therefore, TGF-β was used as an inducer of WPMY-1 proliferation. Our results demonstrated that TGF-β at a concentration of 5 ng/mL significantly promoted WPMY-1 proliferation. This correlated with a previous study that reported that 5 ng/mL TGF-β induces the myofibroblast phenotype of WPMY-1 cells [33]. Interestingly, TGF-β-induced WPMY-1 cell proliferation was reduced by treatment with the ethanolic extract of *C. latifolium* leaves (Fig. 2). To create reproducible results and investigate the chemical composition of this extract, which may be related to its antiproliferative activity, standardization of the *C. latifolium* ethanolic leaf extract was further carried out.

Generally, herbal plant extracts are superior to mainstream medicines in certain aspects, i.e., they are easy to use and inexpensive, require less effort and time, and are sometimes more effective than a single drug [25]. However, the weakness of using herbal medicines is their inconsistent in quality and potency. Due to the variations in the chemical compositions and efficacies of herbal extracts, standardization can ensure their quality, consistency, and safety [34]. The use of the main components of herbal medicines as chemical markers is a method of quality control because the major components show more consistency than the minor components of plant extracts and are easy to detect and quantify [27]. Previous reports have revealed that plants in the genus *Crinum*, including *C. latifolium*, are rich in alkaloids [35]. Therefore, two major alkaloids were isolated (Fig. 3) and used as chemical markers for *C. latifolium* extract standardization. The simultaneous analysis of two components could provide more benefits than relying on only a single component. For instance, the parts of a plant collected, the collection time and the extraction methods utilized could affect the amounts of chemical markers. If the amount of one marker is inconclusive, a different marker can still be quantified and indicate the quality of the plant material. In addition, the ratio of the two major components obtained from the analysis could be a factor in estimating the quality of the plant samples. For the reasons described earlier, a UHPLC method was developed and validated to quantify (1) and (2). The developed method showed good linearity, quantification range and precision and reasonable accuracy.

As previously mentioned, many standardization protocols choose major components as chemical markers. However, most of these compounds show no related biological activity and fail to reflect the efficacy of the plant extract [27]. Therefore, we wanted to determine whether the major alkaloid components (1) and (2) possess antiproliferative activity. Surprisingly, the antiproliferative assay showed that (1) and (2) significantly decreased the proliferation of TGF-β-treated WPMY-1 cells. This confirmed the antiproliferative activity of major alkaloids (1) and (2) (Fig. 6). Thus, (1) and (2) could be used as bioactive markers for *C. latifolium* leaf extracts. However, the use of (1) and (2) as bioactive markers is limited because the antiproliferative effect of the *C. latifolium* leaf extract might receive contributions from other constituents.

Many studies have revealed synergistic, additive, and antagonistic effects from the constituents in plant extracts. For instance, several major components in *Artemisia annua* L. were proven to have synergistic or antagonistic effects on the biological activity of the plant extract [36–39]. Another example was *Echinacea purpurea* L. extract, in which the polysaccharide components showed a synergistic effect on immunostimulant activity [40, 41]. According to the examples above, we suspected that the combination of (1) and (2) from the *C. latifolium* leaf extract could affect the efficacy of the extract*.* From the drug combination results, (1) was more potent than (2), but the maximum efficacy of (1) was limited at a concentration of 3 ng/mL (Fig. 7A). This finding was related to the interaction landscape results, in which a synergistic effect was found within the region where the dose of (1) was 0–3 ng/mL (Fig. 7B). This result also indicated that the decent level of (1) could enhance the combination effect to some extent. In the ZIP model, a synergistic score lower than 0 indicates antagonism, a score from 0 to 10 specifies an additive effect, and a score greater than 10 shows synergism. In this study, the summary ZIP score was 6.968; thus, the overall combination effect of (1) and (2) was additive. According to the findings, the coexistence of (1) and (2) and their ratio could benefit the effectiveness of the *C. latifolium* leaf extract to inhibit TGF-β-induced WPMY-1 proliferation. Moreover, these two major isolated alkaloids could be used as bioactive markers for *C. latifolium* leaf extract standardization. In addition, the ratio of these two major isolated alkaloids was 1:1, and this value could be used for quality control of *C. latifolium* leaf extracts with an anti-BPH effect during drug manufacturing. However, the molecular mechanism of the extract in the treatment of BPH needs to be further investigated.

## Conclusion

*C. latifolium* leaf extract was proven for the first time to possess an antiproliferative effect on TGF-β-treated WPMY-1 cells. The ethanolic extract of *C. latifolium* leaves was successfully standardized by a validated UHPLC method using two major alkaloids, lycorine (1) and 6α-hydroxybuphanidrine (2). Both alkaloids showed antiproliferative effects against TGF-β-treated WPMY-1 cells, indicating their potential as bioactive markers for *C. latifolium* leaf extract quality control. The drug combination study revealed an additive effect of (1) and (2). This study confirms the anti-BPH activity of *C. latifolium* according to its traditional use and discloses the relevance between the quantity of the major alkaloids and the antiproliferative activity of the extract. The present work will benefit the quality assessment and standardization of *C. latifolium* raw materials, extracts, and herbal products containing Amaryllidaceae alkaloids. However, the underlying molecular mechanism of the *C. latifolium* extract and its alkaloids on BPH needs to be elucidated.

## Supplementary Information

**Additional file 1:** **Fig. S1** Cell viability after treatment with mitomycin C for 72 h. Data are expressed as the means±SD (***P<0.001). **Fig. S2** Cell viability after treatment with the C. latifolium extract for 72 h. Data are expressed as the means±SD (**P<0.01). **Fig. S3** Proliferation of WPMY-1 cells treated with TGF-β. Data are expressed as the means±SD (*P< 0.05 and ***P<0.001). **Fig. S4** UV spectrum of lycorine in EtOH. **Fig. S5** High-resolution mass spectrum of lycorine. **Fig. S6** 1H-NMR spectrum (400 MHz) of lycorine in DMSO-d6. **Fig. S7** 13C-NMR spectrum (100 MHz) of lycorine in DMSO-d6. **Fig. S8** High-resolution mass spectrum of 6α-hydroxybuphanidrine. **Fig. S9** UV spectrum of 6α-hydroxybuphanidrine in EtOH. **Fig. S10** 400 MHz 1H-NMR spectrum (400 MHz) of 6α-hydroxybuphanidrine in CDCl3. The peak at 2.17 ppm is a trace signal from acetone. **Fig. S11** 13C-NMR spectrum (100 MHz) of 6α-hydroxybuphanidrine in CDCl3. The peak at 30.95 ppm is a trace signal from acetone. **Fig. S12** Cell viability after treatment with lycorine for 72 h. Data are expressed as the means±SD (***P<0.001). **Fig. S13** Cell viability after treatment with 6α-hydroxybuphanidrine for 72 h. Data are expressed as means±SD (***P<0.001).

## Data Availability

The data used to support the findings of this study are included within the article.

## Abbreviations

ACS: American Chemical Society
ANOVA: Analysis of variance
AR: Analytical reagent
ATCC: American Type Culture Collection
BPH: Benign prostatic hyperplasia
^13^C-NMR: Carbon nuclear magnetic resonance
CO_2_: Carbon dioxide
COSY: Correlation spectroscopy
DMSO: Dimethyl sulfoxide
EtOAc: Ethyl acetate
EtOH: Ethanol
HMBC: Heteronuclear multiple bond correlation
^1^H-NMR: Proton nuclear magnetic resonance
HPLC: High-performance liquid chromatography
HPTLC: High-performance thin-layer chromatography
HRTOFMS: High-resolution time-of-flight mass spectrometry
H_2_SO_4_: Sulfuric acid
HSQC: Heteronuclear single quantum coherence
ICH: International Council for Harmonisation
LOD: Limit of detection
LOQ: Limit of quantitation
MeOH: Methanol
MTT: 3-(4,5-Dimethylthiazol-2-yl)-2,5-diphenyltetra-sodium bromide
NOESY: Nuclear Overhauser effect spectroscopy
PTFE: Polytetrafluoroethylene
R^2^: Correlation coefficient
%RSD: Relative standard deviation
SD: Standard deviation
SiO_2_: Silica gel
TGF-β: Transforming growth factor-beta
TLC: Thin-layer chromatography
UHPLC: Ultrahigh-performance liquid chromatography
UV: Ultraviolet spectroscopy
ZIP: Zero interaction potency
