# Supplementary material for: Standardization of the ethanolic extract of Crinum latifolium leaves by two bioactive markers with antiproliferative activity against TGF-β-promoted prostate stromal cells (WPMY-1)
Source: BMC Complement Med Ther. 2022 May 18;22:139. doi: 10.1186/s12906-022-03617-x (PMC9118764; doi:10.1186/s12906-022-03617-x)
Supplement: Supplementary file 1 — Additional file 1: Fig. S1 Cell viability after treatment with mitomycin C for 72 h. Data are expressed as the means±SD (***P<0.001). Fig. S2 Cell viability after treatment with the C. latifolium extract for 72 h. Data are expressed as the means±SD (**P<0.01). Fig. S3 Proliferation of WPMY-1 cells treated with TGF-β. Data are expressed as the means±SD (*P< 0.05 and ***P<0.001). Fig. S4 UV spectrum of lycorine in EtOH. Fig. S5 High-resolution mass spectrum of lycorine. Fig. S6 1H-NMR spectrum (400 MHz) of lycorine in DMSO-d6. Fig. S7 13C-NMR spectrum (100 MHz) of lycorine in DMSO-d6. Fig. S8 High-resolution mass spectrum of 6α-hydroxybuphanidrine. Fig. S9 UV spectrum of 6α-hydroxybuphanidrine in EtOH. Fig. S10 400 MHz 1H-NMR spectrum (400 MHz) of 6α-hydroxybuphanidrine in CDCl3. The peak at 2.17 ppm is a trace signal from acetone. Fig. S11 13C-NMR spectrum (100 MHz) of 6α-hydroxybuphanidrine in CDCl3. The peak at 30.95 ppm is a trace signal from acetone. Fig. S12 Cell viability after treatment with lycorine for 72 h. Data are expressed as the means±SD (***P<0.001). Fig. S13 Cell viability after treatment with 6α-hydroxybuphanidrine for 72 h. Data are expressed as means±SD (***P<0.001). [file 12906_2022_3617_MOESM1_ESM.docx]

**Supplementary lnformation**

**Standardization of the ethanolic extract of *Crinum latifolium* leaves by two bioactive markers with antiproliferation activity on TGF-β-promoted prostate stromal cells (WPMY-1)**

**
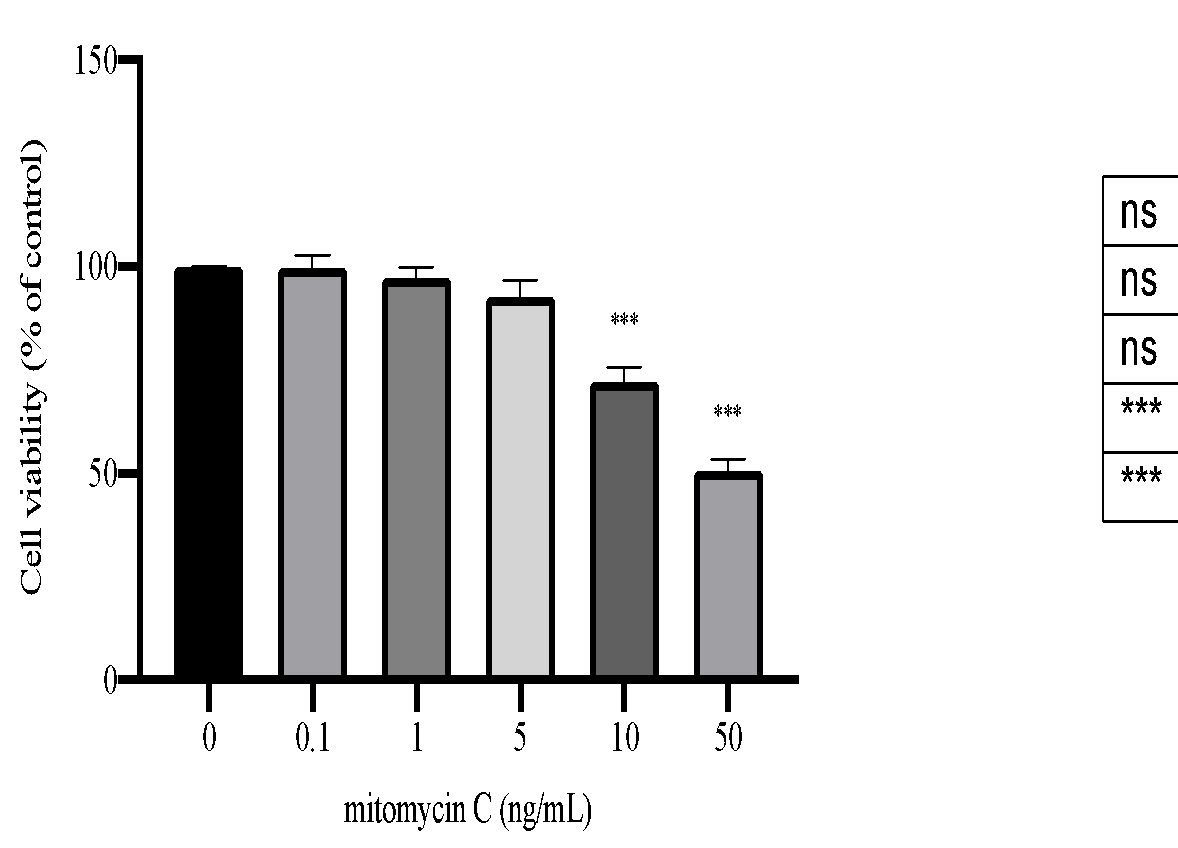
**

**Fig. S1** Cell viability after treatment with mitomycin C for 72 h. Data are expressed as the means±SD (****P*<0.001).

**
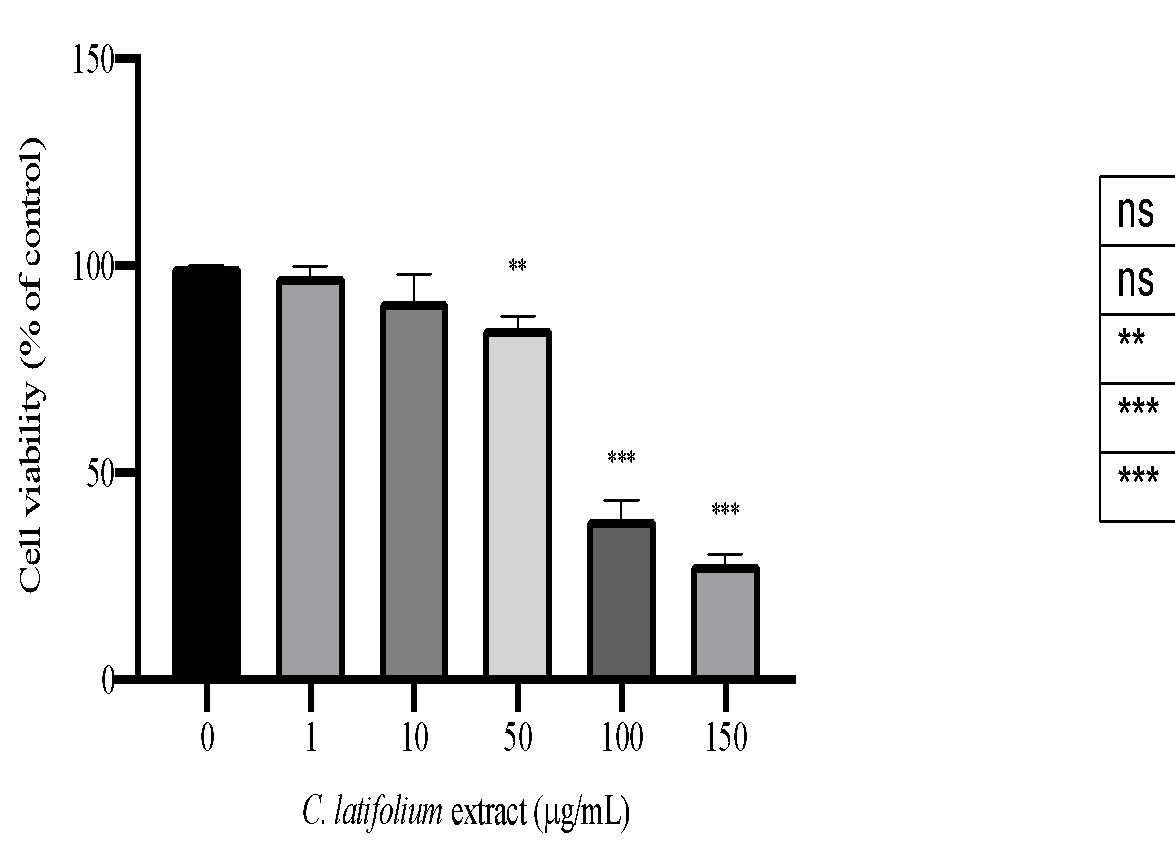
**

**Fig. S2** Cell viability after treatment with the *C. latifolium* extract for 72 h. Data are expressed as the means±SD (***P*<0.01).

**
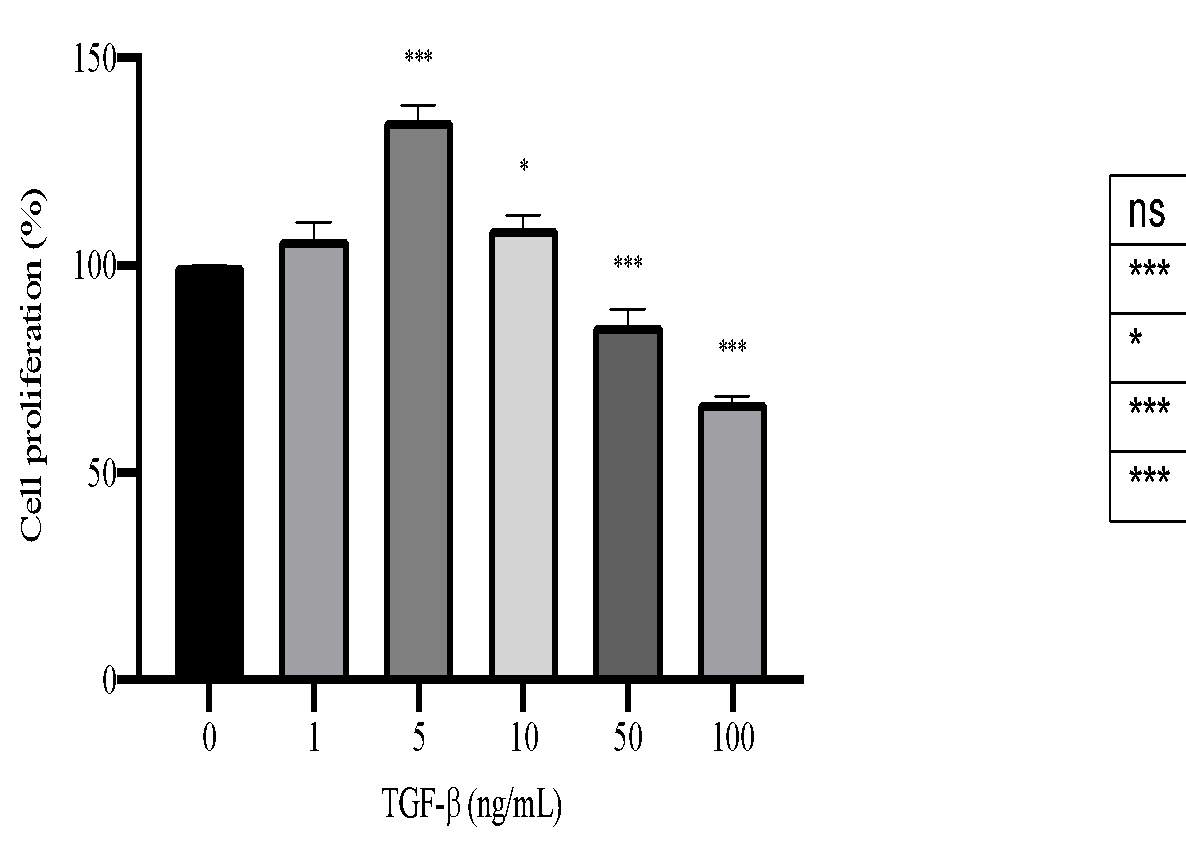
**

**Fig. S3** Proliferation of WPMY-1 cells treated with TGF-β. Data are expressed as the means±SD (**P*< 0.05 and ****P*<0.001).

**
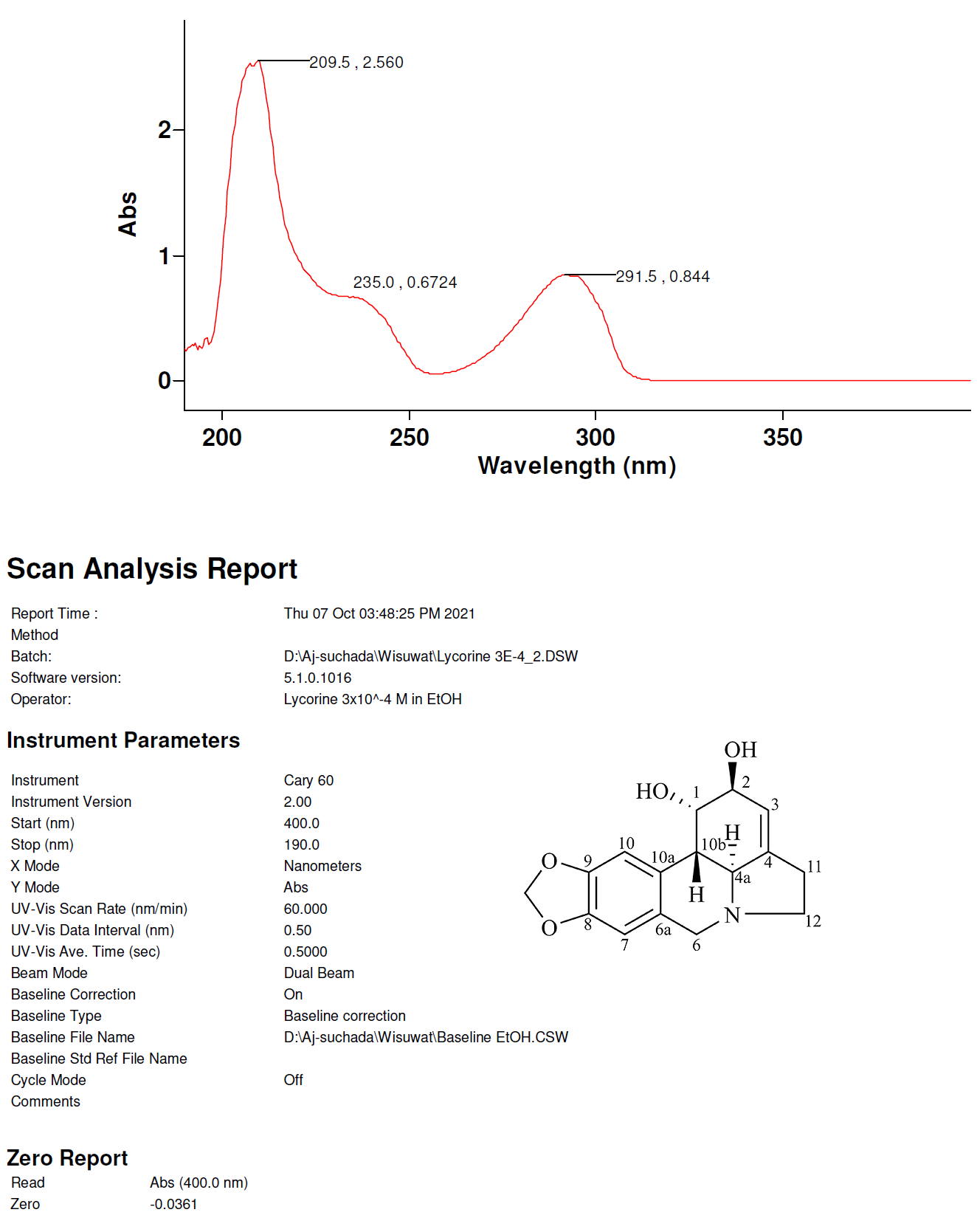
**

**Fig. S4** UV spectrum of lycorine in EtOH.

**
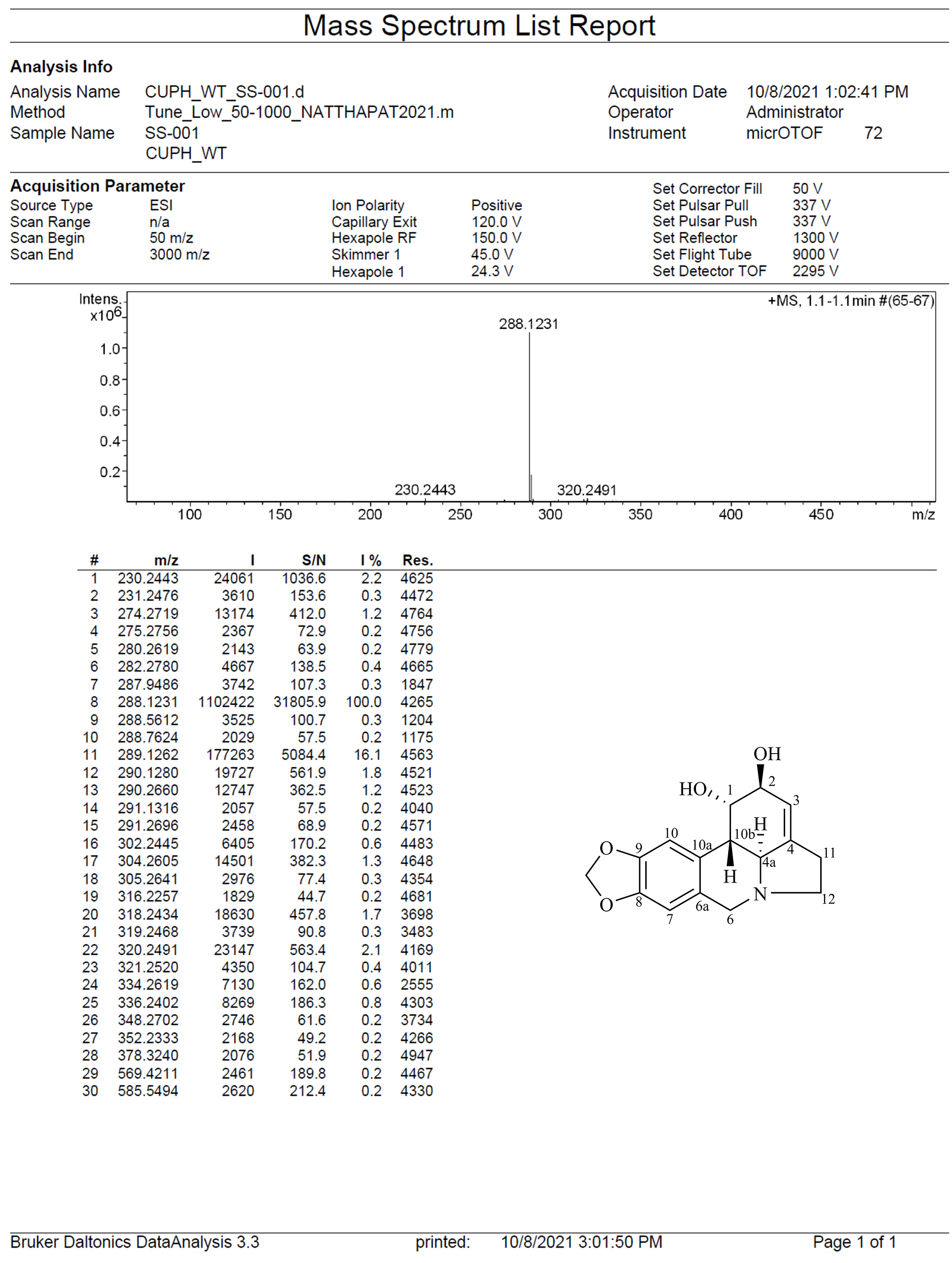
**

**Fig. S5** High-resolution mass spectrum of lycorine.


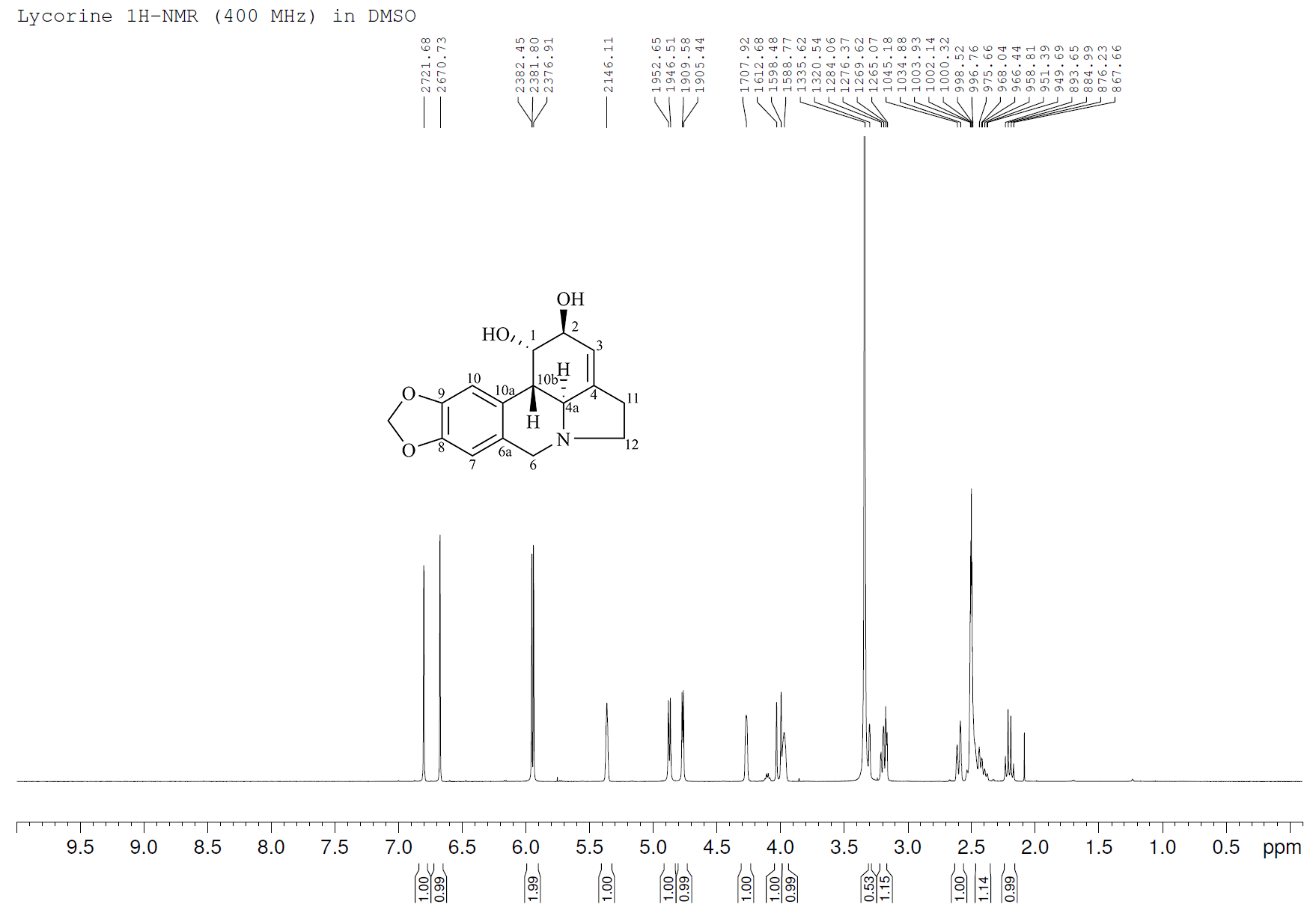


**Fig. S6** ^1^H-NMR spectrum (400 MHz) of lycorine in DMSO-d6.


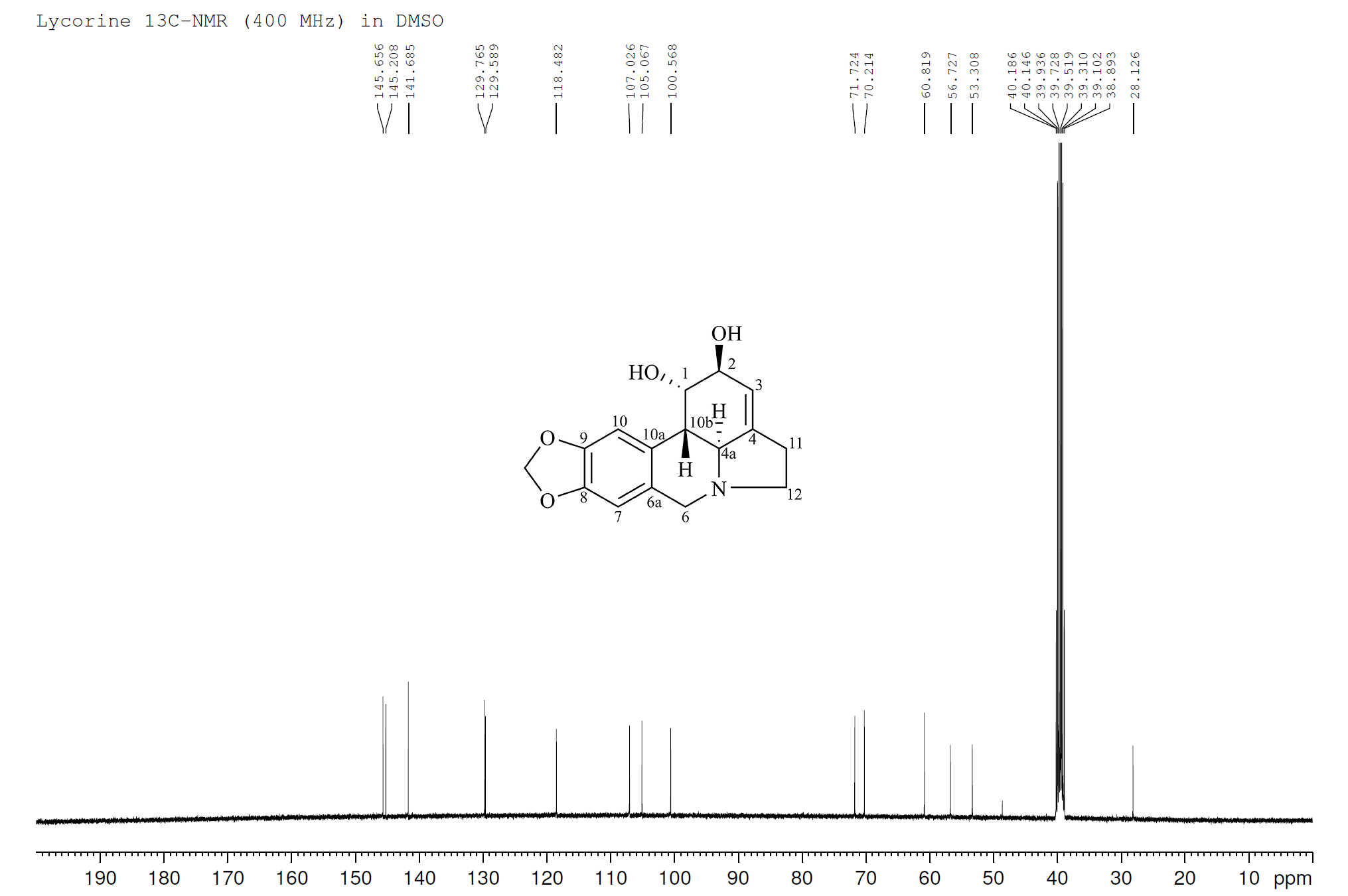


**Fig. S7** ^13^C-NMR spectrum (100 MHz) of lycorine in DMSO-d6.

**
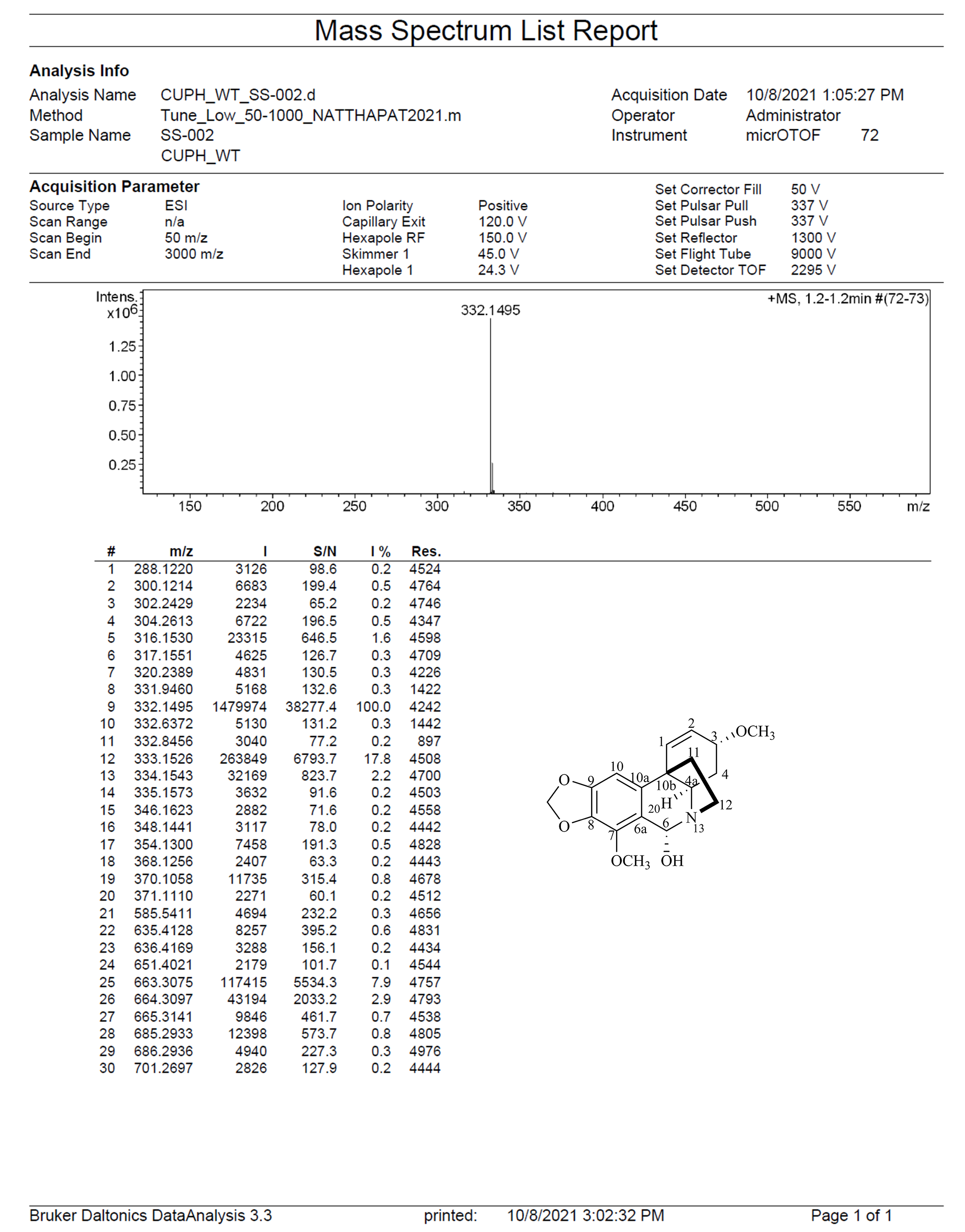
**

**Fig. S8** High-resolution mass spectrum of 6α-hydroxybuphanidrine.

**
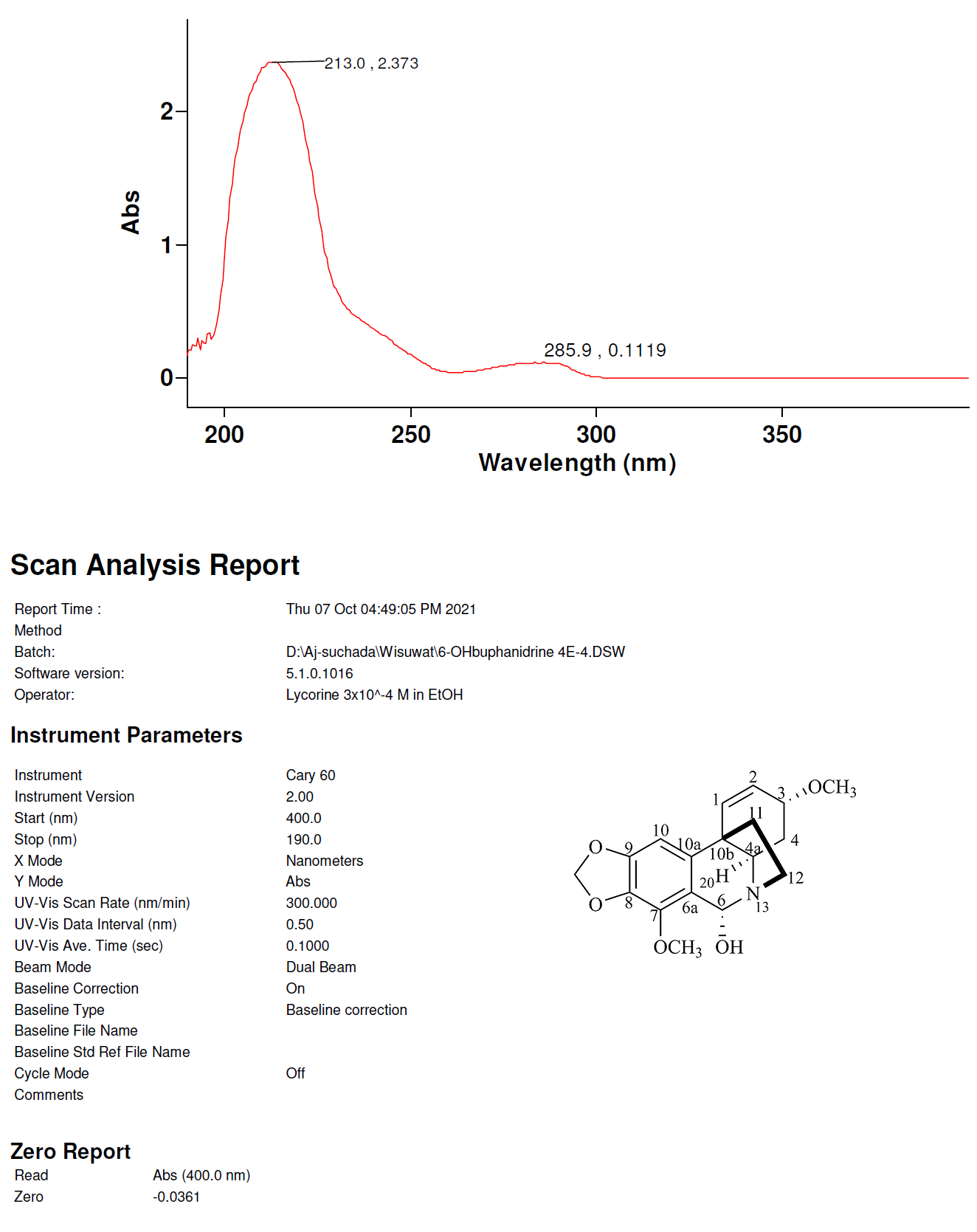
**

**Fig. S9** UV spectrum of 6α-hydroxybuphanidrine in EtOH.


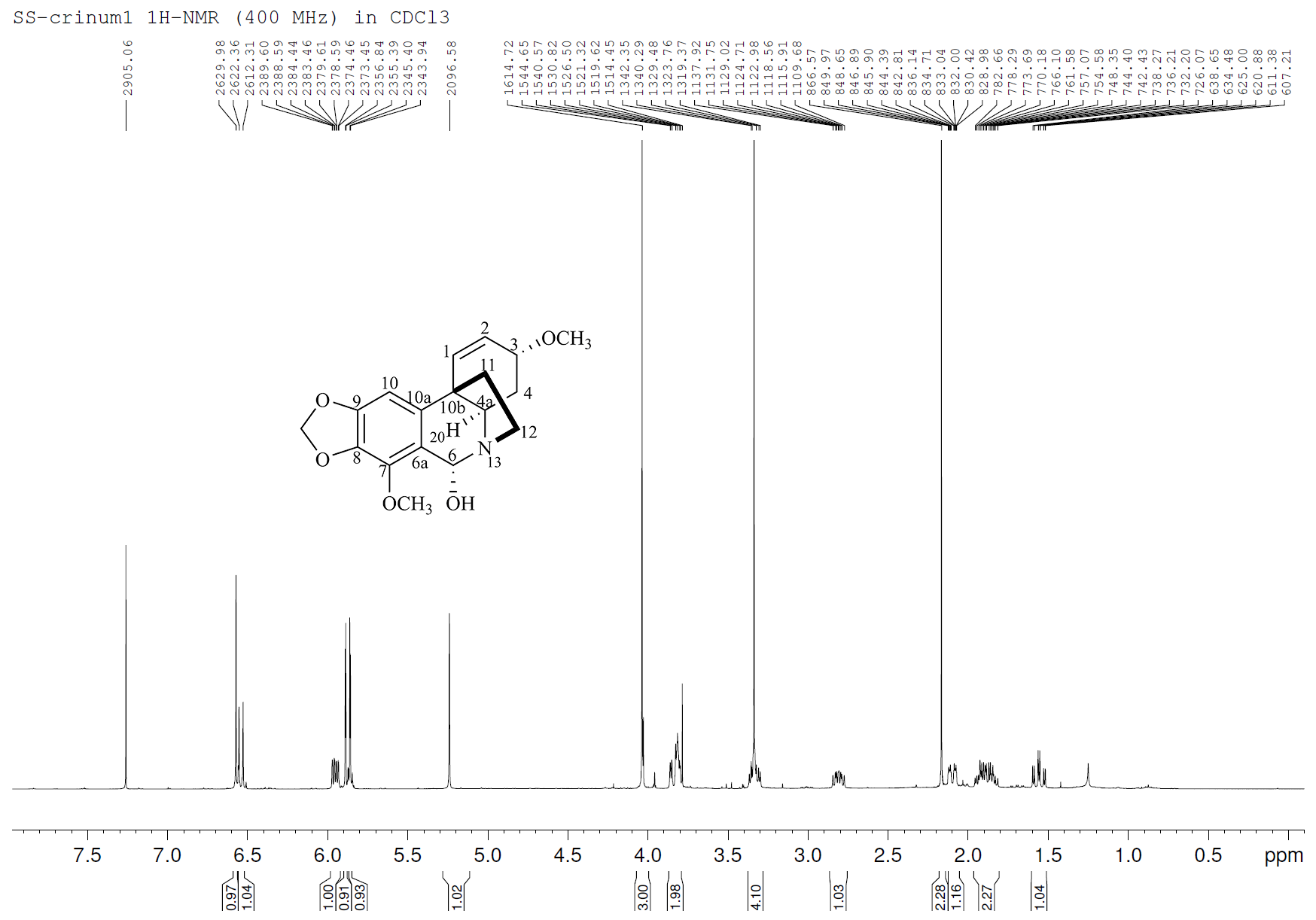


**Fig. S10** 400 MHz ^1^H-NMR spectrum (400 MHz) of 6α-hydroxybuphanidrine in CDCl_3_. The peak at 2.17 ppm is a trace signal from acetone.

**
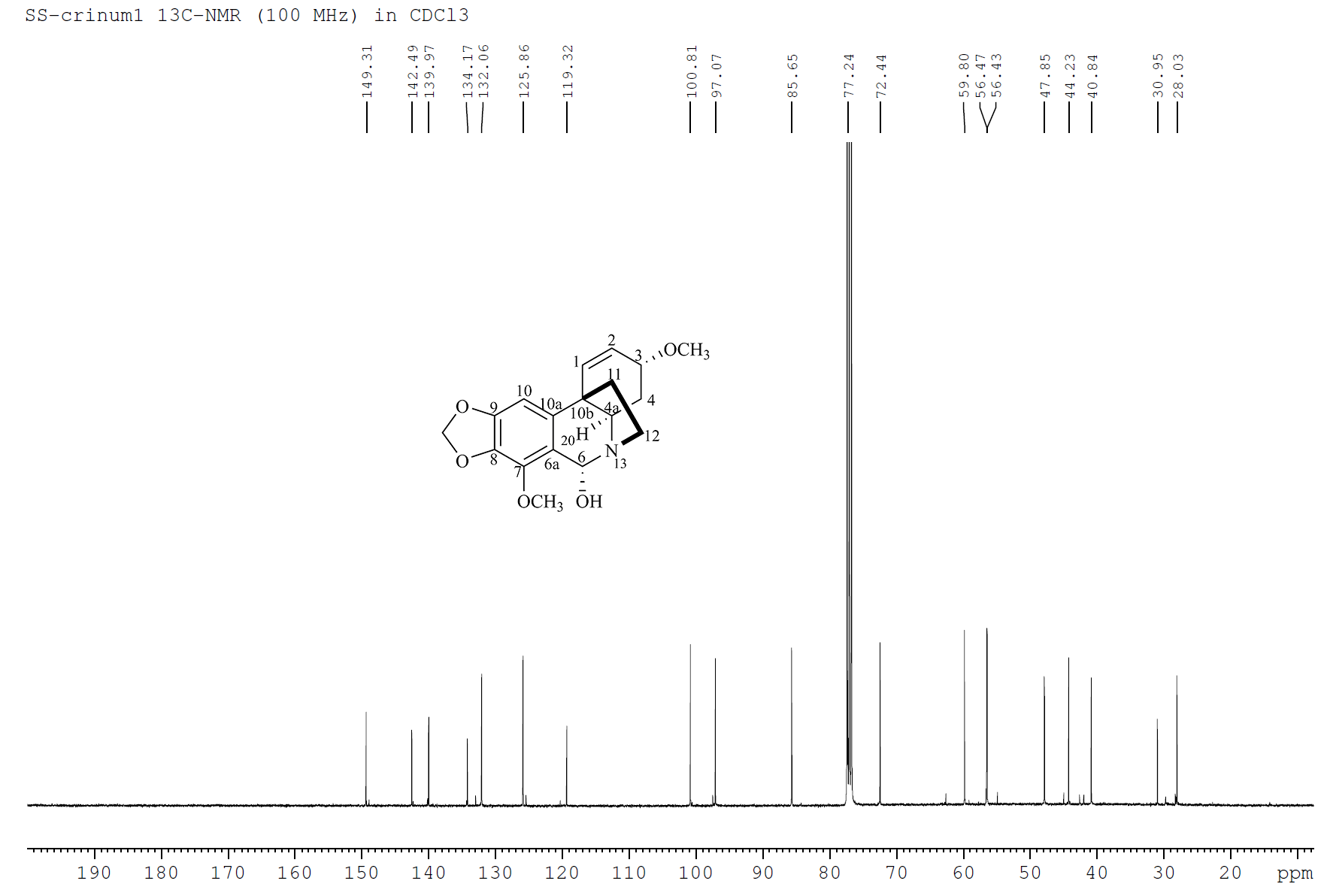
**

**Fig. S11** ^13^C-NMR spectrum (100 MHz) of 6α-hydroxybuphanidrine in CDCl_3_. The peak at 30.95 ppm is a trace signal from acetone.

**
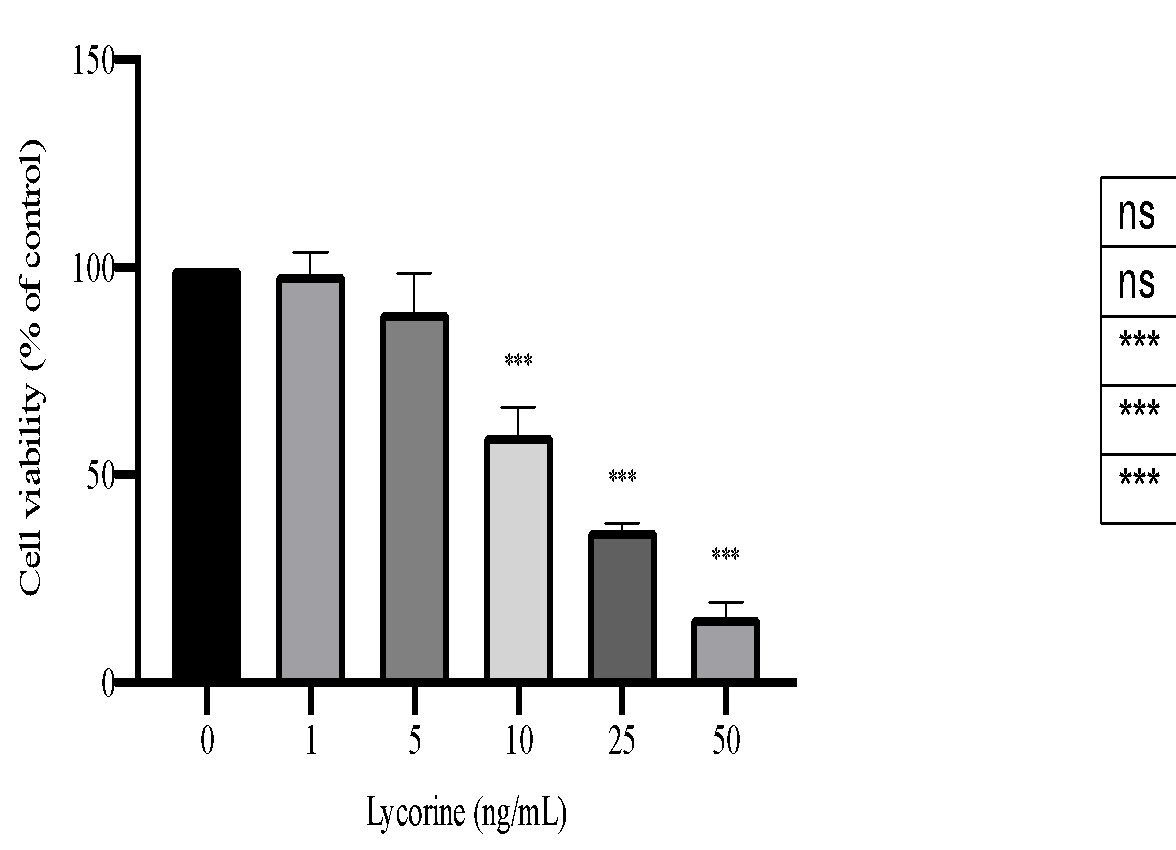
**

**Fig. S12** Cell viability after treatment with lycorine for 72 h. Data are expressed as the means±SD (****P*<0.001).

**
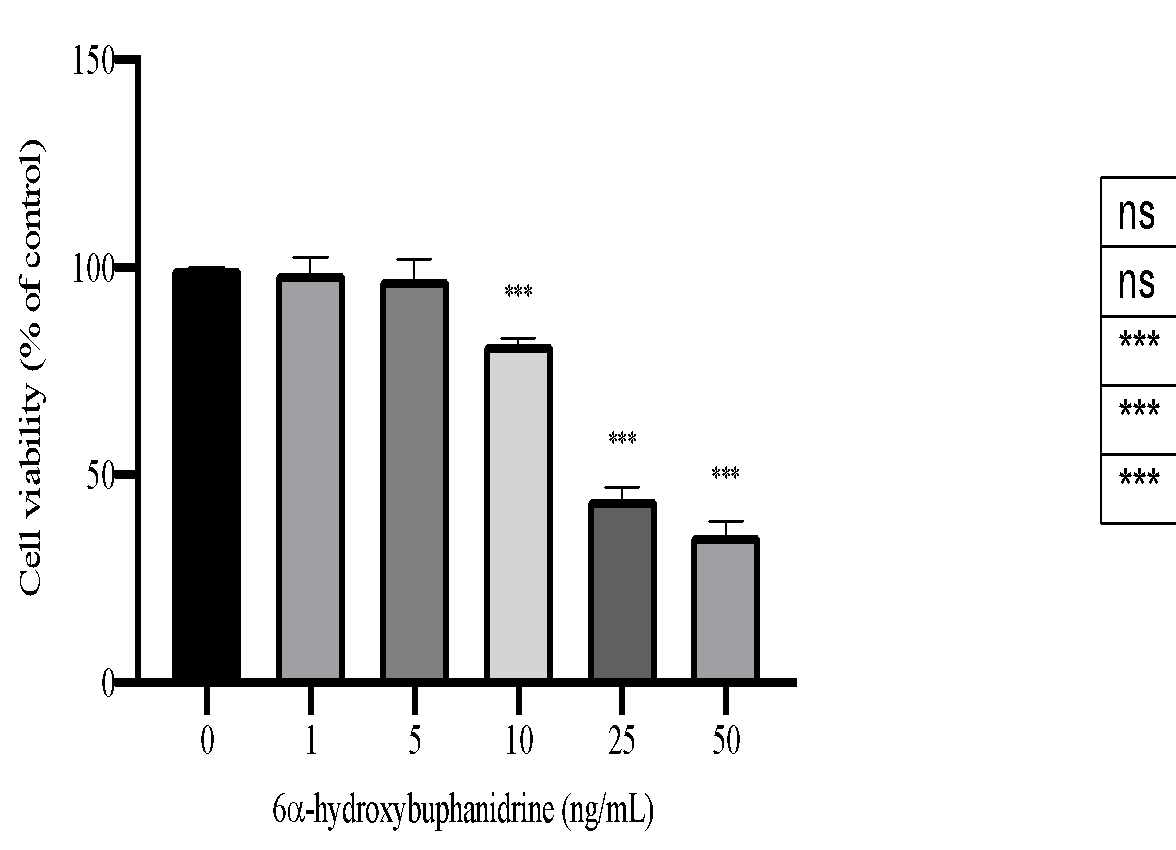
**

**Fig. S13** Cell viability after treatment with 6α-hydroxybuphanidrine for 72 h. Data are expressed as means±SD (****P*<0.001).
